# Bioceramics Based on β-Calcium Pyrophosphate

**DOI:** 10.3390/ma15093105

**Published:** 2022-04-25

**Authors:** Tatiana Safronova, Andrey Kiselev, Irina Selezneva, Tatiana Shatalova, Yulia Lukina, Yaroslav Filippov, Otabek Toshev, Snezhana Tikhonova, Olga Antonova, Alexander Knotko

**Affiliations:** 1 Department of Chemistry, Lomonosov Moscow State University, Building, 3, Leninskie Gory, 1, 119991 Moscow, Russia; artes915@yandex.ru (A.K.); shatalovatb@gmail.com (T.S.); 2 Department of Materials Science, Lomonosov Moscow State University, Building, 73, Leninskie Gory, 1, 119991 Moscow, Russia; filippovyy@my.msu.ru (Y.F.); otabektoshev0995@mail.ru (O.T.); kurbatova.snezhana@yandex.ru (S.T.); knotko@inorg.chem.msu.ru (A.K.); 3 Institute of Theoretical and Experimental Biophysics, Russian Academy of Sciences, Institutskaya, 3, 142290 Pushchino, Russia; selezneva_i@mail.ru; 4 Priorov National Medical Research Center of Traumatology and Orthopedics, Priorova, 10, 127299 Moscow, Russia; lukinayus@cito-priorov.ru; 5 Department of Engineering Design of Technological Equipment, Mendeleev University of Chemical Technology, Miusskaya, 9, 125047 Moscow, Russia; 6 Research Institute of Mechanics, Lomonosov Moscow State University, Michurinsky, 1, 119192 Moscow, Russia; 7 Baikov Institute of Metallurgy and Material Science, Russian Academy of Sciences, Leninsky, 49, 119334 Moscow, Russia; oantonova@imet.ac.ru

**Keywords:** calcium lactate pentahydrate, monocalcium phosphate monohydrate, mechanical activation, powder, brushite, monetite, calcium pyrophosphate, ceramics, biocompatibility

## Abstract

Ceramic samples based on β-calcium pyrophosphate β-Ca_2_P_2_O_7_ were prepared from powders of γ-calcium pyrophosphate γ-Ca_2_P_2_O_7_ with preset molar ratios Ca/P = 1, 0.975 and 0.95 using firing at 900, 1000, and 1100 °C. Calcium lactate pentahydrate Ca(C_3_H_5_O_3_)_2_⋅5H_2_O and monocalcium phosphate monohydrate Ca(H_2_PO_4_)_2_⋅H_2_O were treated in an aqua medium in mechanical activation conditions to prepare powder mixtures with preset molar ratios Ca/P containing calcium hydrophosphates with Ca/P = 1 (precursors of calcium pyrophosphate Ca_2_P_2_O_7_). These powder mixtures containing calcium hydrophosphates with Ca/P = 1 and non-reacted starting salts were heat-treated at 600 °C after drying and disaggregation in acetone. Phase composition of all powder mixtures after heat treatment at 600 °C was presented by γ-calcium pyrophosphate γ-Ca_2_P_2_O_7_ according to the XRD data. The addition of more excess of monocalcium phosphate monohydrate Ca(H_2_PO_4_)_2_·H_2_O (with appropriate molar ratio of Ca/P = 1) to the mixture of starting components resulted in lower dimensions of γ-calcium pyrophosphate (γ-Ca_2_P_2_O_7_) individual particles. The grain size of ceramics increased both with the growth in firing temperature and with decreasing molar ratio Ca/P of powder mixtures. Calcium polyphosphate (t _melt_ = 984 °C), formed from monocalcium phosphate monohydrate Ca(H_2_PO_4_)_2_⋅H_2_O, acted similar to a liquid phase sintering additive. It was confirmed by tests in vitro that prepared ceramic materials with preset molar ratios Ca/P = 1, 0.975, and 0.95 and phase composition presented by β-calcium pyrophosphate β-Ca_2_P_2_O_7_ were biocompatible and could maintain bone cells proliferation.

## 1. Introduction

Ceramics based on calcium phosphates are widely used for bone defect treatment [1,2]. Resorbable calcium phosphate ceramic materials are necessary for the implementation of bone defect treating methods of regenerative medicine [3]. It is known from the scientific literature that the ability of inorganic materials to resorb is connected with the ability to solve in an aqua medium [4]; this ability to a great extent depends on the crystal structure of an inorganic substance [5]. Calcium phosphate’s ability to solve in an aqua medium can be enhanced by decreasing Ca/P molar ratio [6]. At the same time pH generated during the dissolution of calcium phosphate has to be close to neutral as a necessary feature of biocompatibility [7]. So, the ceramics based on calcium pyrophosphate Ca_2_P_2_O_7_ can be an interesting object of investigation both due to molar ratio Ca/P = 1 (it is less than molar ratio Ca/P = 1.67 for insoluble hydroxyapatite Ca_10_(PO_4_)_6_(OH)_2_ and Ca/P = 1.5 for tricalcium phosphate Ca_3_(PO_4_)_2_) and due to pH close to neutral (pH~7) during immersion to the water [8].

According to the information from scientific literature ceramics based on β-calcium pyrophosphate or ceramics containing phase of β-calcium pyrophosphate have repeatedly been the subject of investigation dealing with creation of materials for bone defect treatment [9,10,11,12,13]. In all these technics different powder precursors were used.

Different powders and powder mixtures with molar ratio Ca/P = 1 can be used as precursors of high-temperature β-modification of calcium pyrophosphate Ca_2_P_2_O_7_ as the ceramic phase. Powders of brushite CaHPO_4_·2H_2_O [9], monetite CaHPO_4_ [10], hydrated amorphous calcium pyrophosphate Ca_2_P_2_O_7_·xH_2_O [11,12], calcium pyrophosphate Ca_2_P_2_O_7_ in forms of γ or β modifications [13,14], can be used as starting direct powder precursors for β-calcium pyrophosphate β-Ca_2_P_2_O_7_ ceramics preparation.

Solid-state sintering of calcium phosphate ceramics has some difficulties due to the complexity of mass-transfer because of the lower diffusion of the large, multiply charged phosphate or pyrophosphate ions [15]. It is impossible to help sintering of calcium pyrophosphate ceramics with elevating of firing temperature because of β-α phase transition at 1150 °C [16]. Using fine powders, special atmospheres of firing, and sintering additives can enhance the sintering ability of any ceramic material. Chemical synthesis of calcium phosphate powders for ceramic preparation is used for enhancement of powder sintering activity [17]. CO_2_ or H_2_O atmosphere can intensify the sintering of hydroxyapatite [18]. Sintering additives with the ability to introduce defects in the crystal structure can help solid-state sintering [19,20]. Quite an ordinary decision to overcome the difficulties in sintering of calcium phosphate ceramics consists in using liquid phase sintering [21]. Liquid phase sintering can be realized when the sintering additive is presented in a quantity of a wide interval from 1% to 40%. Sodium phosphates [22,23], sodium carbonate [24], sodium/potassium nitrates [25], and calcium polyphosphate [9,26,27] were used as sintering additives for calcium pyrophosphate ceramic preparation. Potassium carbonate [28,29], potassium chloride [30], and calcium chloride [31] used as sintering additives for ceramic based on hydroxyapatite can also be used as sintering additives for calcium pyrophosphate ceramics. It should be noted, that in investigations [26,27], calcium polyphosphate was introduced in preceramic samples prepared as cement stone via excess of monocalcium phosphate monohydrate. Application of low temperature melting salts with biocompatible cations such as potassium or sodium has a slight disadvantage, which consists in the possibility of the drift of phase composition of bioceramics to the oxide systems Na_2_O-CaO-P_2_O_5_ or K_2_O-CaO-P_2_O_5_ and the formation of phases of double phosphates due to heterophase reactions. The possibility of these reactions can lead to the diminution of the quantity of sintering additive when processing ceramics, and then formation in ceramics of those phases of double phosphates, which, in case of notable amount, can generate basic pH harmful for a patient organism if implanted. Ceramic materials in the CaO-P_2_O_5_ system with low content of Ca(PO_3_)_2_ as expected will be more friendly to the living organism if implanted.

As precursors of the calcium polyphosphate phase in ceramics, the different compounds can be used [32]. The following compounds with molar ratio Ca/P = 0.5 also can be used as precursors of calcium polyphosphate: amorphous hydrated calcium polyphosphate Ca(PO_3_)_2_·xH_2_O [33], CaH_2_(HPO_3_)_2_ [34], CaH_2_P_2_O_7_ [35], CaH_2_P_2_O_7_·H_2_O [36], CaNH_4_HP_2_O_7_ [37,38], Ca(NH_4_)_2_P_2_O_7_·H_2_O [39,40], CaNH_4_HP_2_O_7,_ Ca_2_NH_4_H_3_(P_2_O_7_)_2_ H_2_O, Ca_2_NH_4_H_3_(P_2_O_7_)_2_·3H_2_O [41], Ca(H_2_PO_4_)_2_, and Ca(H_2_PO_4_)_2_·H_2_O [42,43].

Earlier, it was shown that the synthesis of fine grain powder of monetite CaHPO_4_ can be carried out in conditions of mechanical activation from powder mixture including hydroxyapatite Ca_10_(PO_4_)_6_(OH)_2_ and monocalcium phosphate monohydrate Ca(H_2_PO_4_)_2_·H_2_O [10]. It also was shown that treatment in a water solution of lactic acid allowed preparing powder of monocalcium phosphate monohydrate Ca(H_2_PO_4_)_2_·H_2_O with lower dimensions of particles as far lactic acid can act as a surface-active substance [44]. It is well known that the smaller the particle dimensions of starting components the more homogeneous powder mixture can be prepared. So, in the present work, powder mixtures for ceramics production were prepared in the first time in conditions of mechanical activation from calcium lactate pentahydrate Ca(C_3_H_5_O_3_)_2_·5H_2_O and monocalcium phosphate monohydrate Ca(H_2_PO_4_)_2_·H_2_O when the last was taken in excess and used also as a precursor of calcium polyphosphate Ca(PO_3_)_2_ which played the role of a liquid phase sintering additive.

The aim of the present work consisted in the preparation of β-calcium pyrophosphate ceramics with the assistance of calcium polyphosphate as a liquid phase sintering additive based on fine synthetic powders of γ-calcium pyrophosphate with Ca/P molar ratios preset as 1, 0.975, 0.95 and investigation of biocompatibility of prepared ceramics in vitro.

## 2. Materials and Methods

### 2.1. Materials

Powders of calcium lactate pentahydrate Ca(C_3_H_5_O_3_)_2_·5H_2_O (CAS no. 814-80-2, food-grade E327 of FCC, Henan Jindan Lactic Acid Technology Co., Ltd., Zhengzhou, Henan, China) and monocalcium phosphate monohydrate Ca(H_2_PO_4_)_2_·H_2_O (CAS no. 10031-30-8, puriss. 99%, Product of Spain, Sigma-Aldrich Chemie GmbH, Steinheim, Germany) were used for powder mixtures preparation.

The target phase compositions of ceramics are shown in Table 1. Calcium polyphosphate was introduced as a sintering additive with a low melting point (984 °C [16]).

Compositions of powder mixtures before treatment in mechanical activation conditions are presented in Table 2.

Reaction (1) corresponding to powder mixture “Pyro” was used to calculate quantities of starting components.

Ca(C_3_H_5_O_3_)_2_⋅5H_2_O + Ca(H_2_PO_4_)_2_⋅H_2_O → 2CaHPO_4_⋅2H_2_O + 2C_3_H_5_O_3_H + 2H_2_O (1)

### 2.2. Powder Mixtures Preparation

A total 10 g of starting components in ratios are presented in Table 2; 50 g of grinding media made from zirconia and 40 mL of distilled water were placed in containers made from zirconia. Containers with starting components were fixed in the planetary mill (Fritch Pulverisette, Idar-Oberstein, Germany). Mechanical activation of suspension initially containing distilled water, calcium lactate pentahydrate Ca(C_3_H_5_O_3_)_2_·5H_2_O, and monocalcium phosphate monohydrate Ca(H_2_PO_4_)_2_·H_2_O was conducted for 15 min at a rotation speed of 600 rpm. Then, powder mixtures after drying for a week were disaggregated in a planetary mill in acetone medium for 15 min at a rotation speed of 600 rpm. After drying powder mixtures were passed through the sieve with 200 mm mesh. Then, powder mixtures were heat-treated at 600 °C for 30 min.

### 2.3. Ceramic Samples Preparation

Powder mixtures prepared as described above were used for ceramics preparation. Powder compacts were pressed at 100 MPa in the form of disks with a diameter of 12 mm and height of 1 mm in steel mold using the manual press (Carver Laboratory Press model C, Fred S. Carver, Inc., Wabash, IN, USA). Then, powder compacts were fired in the air at 900, 1000, and 1100 °C with a heating rate of 5 °C/min and 2 h holding at a specified temperature.

### 2.4. Characterization Methods

The phase composition of the prepared powder mixtures, powders after heat treatment at 600 °C, and ceramic samples after firing was determined by X-ray powder diffraction (XRD) analysis using Rigaku D/Max-2500 diffractometer (Rigaku Corporation, Tokyo, Japan) with a rotating anode (Cu-Ka radiation), angle interval 2Ѳ: from 2° to 70°, step 2Ѳ − 0.02°). Phase analysis was performed using the ICDD PDF2 database [45]

Thermal analysis (TA) was performed to determine the total mass loss of the powder mixtures at heating up to 1000 °C in the air using NETZSCH STA 449 F3 Jupiter thermal analyzer (NETZSCH, Selb, Germany). The gas-phase composition was monitored by the quadrupole mass spectrometer QMS 403 Quadro (NETZSCH, Selb, Germany) combined with a thermal analyzer NETZSCH STA 449 F3 Jupiter. The mass spectra (MS) were registered for the following *m/z* values: 18 (H_2_O); 44 (CO_2_); the heating rate was 10 °C/min.

Powders after heat treatment at 600 °C and ceramics after firing were examined by scanning electron microscopy (SEM) on a LEO SUPRA 50VP electron microscope (Carl Zeiss, Jena, Germany; auto-emission source). This investigation was carried out at an accelerating voltage of 3–20 kV using SE2 detectors. The surface of the ceramic samples and powders was coated with a layer of chromium (up to 10 nm).

### 2.5. Biocompatibility Estimation

Ceramic samples fired at 1100 °C were used for the investigation of biocompatibility in vitro.

Primary dental pulp stem cells (cell culture) were used to study the biocompatibility of the prepared ceramics. The dental pulp stem cells culture was obtained from freshly extracted third molar teeth (donor age, 16 years) with a root at least two-thirds formed, which were extracted for orthodontics reasons [46]. The cell cultures were maintained in DMEM/F12 medium supplemented with 10% FBS, 100 units mL^−1^ penicillin, and 100 mg mL^−1^ streptomycin under an 80% humidity with 5% CO_2_ atmosphere at 37 °C.

For assessing cytotoxicity of ceramics direct contact method was used. The samples were placed onto 24-well culture plates. The cells were seeded on the surfaces of ceramic samples at 40,000 cell cm^−2^ and cultured in DMEM/F12 (1:1) medium supplemented with 10% FBS, 100 units mL^−1^ penicillin, and 100 mg mL^−1^ streptomycin at 80% humidity in a 5% CO_2_ atmosphere at 37 °C. The cytotoxicity of the ceramic samples was estimated by evaluating the cell viability through a double-staining fluorescence assay in a direct contact procedure 2 and 7 days after the beginning of experiments. In this study, the ability of the prepared ceramics to support the adhesion of the primary dental pulp stem cells and to stimulate their proliferation was also examined. We used a double-staining assay with SYTO9 (green fluorescent nucleic acid stain), which stains all cells, and propidium iodide (red fluorescent nucleic acid stain), which stains the nuclei of dead cells (L-7007 LIVE/DEAD Bac Light Bacterial Viability Kit, Invitrogen, Thermo Fisher Scientific, Eugene, USA). The cells were visualized using fluorescence microscopy (Axiovert 200, Zeiss, Germany).

The cell-containing surfaces of prepared ceramic specimens after primary dental pulp stem cells cultivation were studied using a Tescan Vega II scanning electron microscope (SEM, Tescan Vega II, Brno, Czech Republic); the imaging was performed in a low vacuum mode at an accelerating voltage of 20 kV (SE detector). To prepare samples of the cell-containing surfaces of ceramics after 2 days of cells cultivation for SEM analysis, the cells were fixed and dehydrated. Briefly, the samples were washed three times with PBS and fixed with glutaraldehyde (2.5% in PBS, pH 7.4) for 2 h. After fixation, the samples were rinsed with PBS once before being dehydrated using a series of solutions. Samples were coated with a thin layer of gold to prevent surface charging (Q150R ES, Quorum Technologies, East Sussex, UK).

The cytotoxicity of the ceramics was evaluated using the MTT test according to ISO 10993-5. The samples were incubated in polypropylene tubes containing DMEM/F12 supplemented with 100 U mL^−1^ penicillin/streptomycin for 3 days at 37 °C under aseptic conditions. In the liquid extracts of materials, the ratio of the mass of the samples (g) to the volume of the culture medium (mL) was 0.1–0.2. DMEM/F12 medium was used as a control. The NCTC L929 cells were used at 40,000 cells cm^−2^ for 24 h before adding the liquid extracts of the material. The extracts were transferred onto a layer of cells and incubated. The viability of the cells was evaluated 1 day after the beginning of experiments by measuring the reduction of the colorless salt tetrazolium(3-[4,5-dimethylthiazol-2-yl]-2,5-diphenyltetrazolium bromide) (MTT) by mitochondrial and cytoplasmic dehydrogenases of living metabolically active cells through the formation of intracellular water-insoluble purple-blue crystals of formazan. The cells were treated with MTT (0.5 mg mL^−1^) at 37 °C for 3 h in air with 5% CO_2_ and 90% humidity. The medium was removed and the formazan was solubilized with 100 µL dimethylsulfoxide (DMSO). The absorption at 540 nm was measured using a microplate spectrophotometer (model 680 BioRad, Bio-Rad Laboratories, Inc., Hercules, CA, USA). The value was an average of three separate experiments. The results for the optical densities were expressed as mean standard deviation. The statistically significant difference between the groups was estimated using the Mann–Whitney U test. Differences at *p* < 0.05 were considered statistically significant.

## 3. Results and Discussion

**Figure 1:**
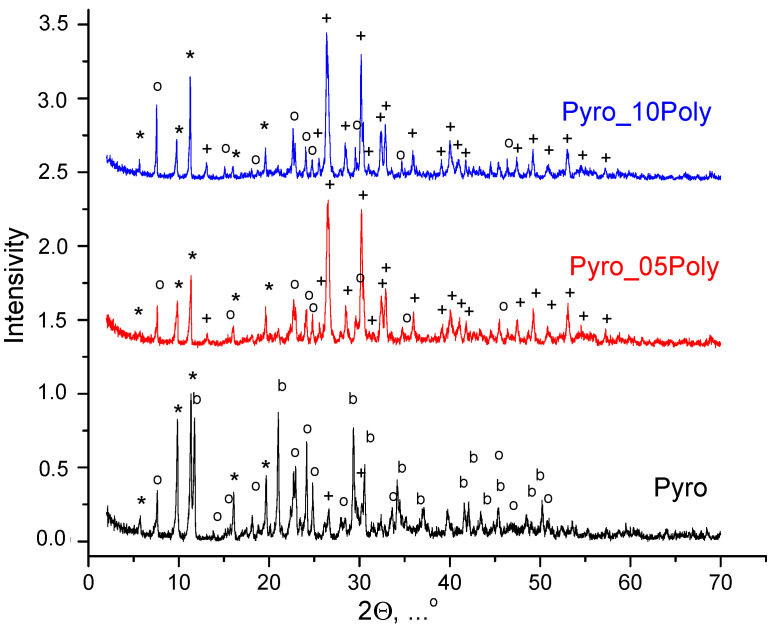
XRD data for powder mixtures prepared in mechanical activation conditions from calcium lactate pentahydrate Ca(C_3_H_5_O_3_)_2_·5H_2_O and monocalcium phosphate monohydrate Ca(H_2_PO_4_)_2_·H_2_O: *—Ca(C_3_H_5_O_3_)_2_·5H_2_O (according to scientific literature data [49,50,51]); o—Ca(H_2_PO_4_)_2_·H_2_O (PDF card 9-347); +—CaHPO_4_ (PDF card 9-80); b—CaHPO_4_·2H_2_O (PDF card 9-77).

According to XRD analysis (Figure 1), after homogenization of starting salts in mechanical activation conditions in a planetary mill in aqua medium powder mixture “Pyro” (Ca/P = 1) included brushite CaHPO_4_·2H_2_O; monetite CaHPO_4_ in small quantity; and starting components, i.e., calcium lactate pentahydrate Ca(C_3_H_5_O_3_)_2_·5H_2_O and monocalcium phosphate monohydrate Ca(H_2_PO_4_)_2_⋅H_2_O. Powder mixtures “Pyro_05Poly” (Ca/P = 0.975) and “Pyro_10Poly” (Ca/P = 0.95) included monetite CaHPO_4_ and starting components, i.e., calcium lactate pentahydrate Ca(C_3_H_5_O_3_)_2_·5H_2_O and monocalcium phosphate monohydrate Ca(H_2_PO_4_)_2_·H_2_O. The presence of starting salts in all powder mixtures under investigation can be explained by the incompleteness of the reactions (1). Moreover, monocalcium phosphate monohydrate Ca(H_2_PO_4_)_2_·H_2_O was intentionally introduced in powder mixtures “Pyro_05Poly” (Ca/P = 0.975) and “Pyro_10Poly” (Ca/P = 0.95) in excess to provide formation of calcium polyphosphate Ca(PO_3_)_2_ at the firing stage. An intentionally introduced excess of monocalcium phosphate monohydrate Ca(H_2_PO_4_)_2_·H_2_O provided more acidic pH of water solution formed during treatment of powder mixtures “Pyro_05Poly” (Ca/P = 0.975) and “Pyro_10Poly” (Ca/P = 0.95) in a planetary mill. More acidic pH of water solution, as shown before in other investigations [47,48], can explain the preferable formation of monetite CaHPO_4_ (calcium hydrophosphate anhydrate) in powder mixtures “Pyro_05Poly” (Ca/P = 0.975) and “Pyro_10Poly” (Ca/P = 0.95) instead of brushite CaHPO_4_·2H_2_O (calcium hydrophosphate dihydrate) as it was for powder mixture “Pyro”.

**Figure 2:**
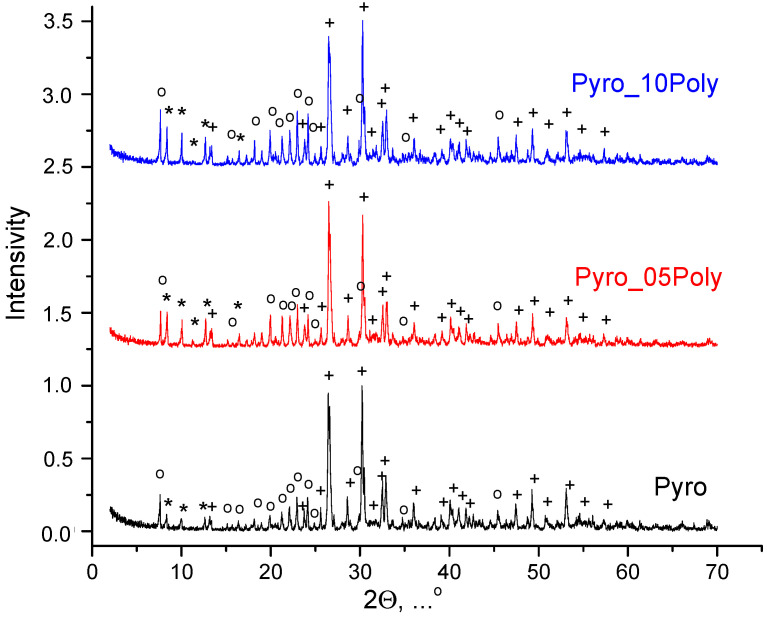
XRD data for powder mixtures prepared in mechanical activation conditions from calcium lactate pentahydrate Ca(C_3_H_5_O_3_)_2_·5H_2_O and monocalcium phosphate monohydrate Ca(H_2_PO_4_)_2_·H_2_O after disaggregation in acetone medium: *—Ca(C_3_H_5_O_3_)_2_·5H_2_O (according scientific literature data [49,50,51]); o—Ca(H_2_PO_4_)_2_·H_2_O (PDF card 9-347); +—CaHPO_4_ (PDF card 9-80).

After drying, the prepared powder mixtures were aggregated to a great extent and the stage of disaggregation was highly necessary. So, after drying powder mixtures were disaggregated in acetone medium in a planetary mill. According to XRD data (Figure 2), after disaggregation in acetone medium in planetary mill, phase composition of all powder mixtures included monetite CaHPO_4_ and starting components, i.e., calcium lactate pentahydrate Ca(C_3_H_5_O_3_)_2_·5H_2_O and monocalcium phosphate monohydrate Ca(H_2_PO_4_)_2_·H_2_O. One can see that the greater the content of monocalcium phosphate monohydrate Ca(H_2_PO_4_)_2_·H_2_O in powder mixture, the more noticeable its main reflex at normalized graphs. Chemical reaction (2) of brushite dehydration taking place during disaggregation in acetone medium in powder mixture “Pyro” is presented below.

CaHPO_4_⋅2H_2_O → CaHPO_4_ + 2H_2_O (2)

**Figure 3:**
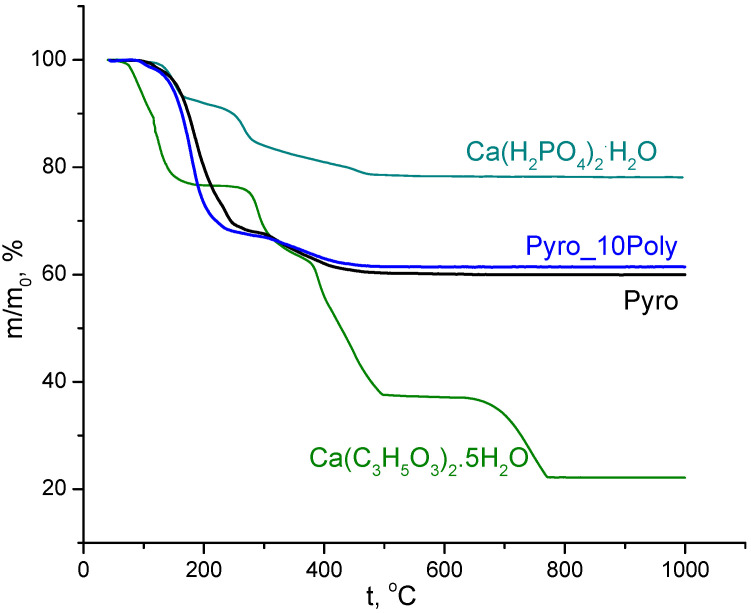
TA of powder mixtures “Pyro” (Ca/P = 1) and “Pyro_10Poly” (Ca/P = 0.95) prepared in mechanical activation conditions from calcium lactate pentahydrate Ca(C_3_H_5_O_3_)_2_·5H_2_O and disaggregated in acetone medium; TA of starting components (calcium lactate pentahydrate Ca(C_3_H_5_O_3_)_2_·5H_2_O and monocalcium phosphate monohydrate Ca(H_2_PO_4_)_2_·H_2_O).

TA data of powder mixtures “Pyro” (Ca/P = 1) and “Pyro_10Poly” (Ca/P = 0.95) prepared in mechanical activation conditions in aqua medium from calcium lactate pentahydrate Ca(C_3_H_5_O_3_)_2_·5H_2_O and monocalcium phosphate monohydrate Ca(H_2_PO_4_)_2_·H_2_O), disaggregated in acetone medium, and TA of starting components (calcium lactate pentahydrate Ca(C_3_H_5_O_3_)_2_·5H_2_O and monocalcium phosphate monohydrate Ca(H_2_PO_4_)_2_·H_2_O) are presented in Figure 3. Total mass loss for powder mixtures “Pyro” (Ca/P = 1) was 40%. Total mass loss for powder mixtures “Pyro_10Poly” (Ca/P = 0.95) was 39%. All processes provided mass loss of the powder mixtures under investigation during heating finished up to 500 °C. As we can assume according to data of XRD analysis of powder mixtures after treatment in acetone medium and the accordant reaction (1), composition of powder mixtures included monetite, lactic acid, and starting components (calcium lactate pentahydrate Ca(C_3_H_5_O_3_)_2_·5H_2_O and monocalcium phosphate monohydrate Ca(H_2_PO_4_)_2_·H_2_O).

So, we can suggest the following reactions that can take place during heating: dehydration of hydrated salts (reactions (3) and (4) [10,52]), decomposition of lactic acid (reaction (5) [53]), formation of calcium pyrophosphate via condensation (6) [10], synthesis of calcium pyrophosphate due to interaction of monocalcium phosphate with calcium lactate (reaction (7)) or due to interaction of monocalcium phosphate monohydrate with calcium lactate pentahydrate (reaction (8)), and formation of calcium polyphosphate due to condensation (reaction (9) [10]).

Ca(H_2_PO_4_)_2_⋅H_2_O = Ca(H_2_PO_4_)_2_ + H_2_O (~200 °C) (3)

Ca(C_3_H_5_O_3_)_2_⋅5H_2_O = Ca(C_3_H_5_O_3_)_2_+5H_2_O (~200 °C) (4)

C_3_H_5_O_3_H+6O_2_ = 3CO_2_ + 3H_2_O (melting ~120 °C) (5)

2CaHPO_4_ = Ca_2_P_2_O_7_ + H_2_O (~300–475 °C) (6)

Ca(H_2_PO_4_)_2_ + Ca(C_3_H_5_O_3_)_2_ + 6O_2_ = Ca_2_P_2_O_7_ + 6CO_2_ + 7H_2_O (~110–500 °C) (7)

Ca(H_2_PO_4_)_2_⋅H_2_O + Ca(C_3_H_5_O_3_)_2_⋅5H_2_O + 6O_2_ = Ca_2_P_2_O_7_ + 6CO_2_ + 13H_2_O (~110–500 °C) (8)

Ca(H_2_PO_4_)_2_ = Ca(PO_3_)_2_ + 2H_2_O (~300–500 °C) (9)

The form of curves m/m_0_ = f(t) in Figure 3 of powder mixtures under investigation are very smooth and differ from curves of starting salts. The smoothness of the lines indicates the possibility of overlapping temperature intervals for listed reactions and their simultaneous occurrence. This difference confirms the possibility both of reactions of condensation (reactions (6) and (9)) and the possibility of formation of calcium pyrophosphate from starting components preserved during treatments in mechanical activation conditions. Differentiation of curves m/m_0_ = f(t) for powder mixtures under investigation allows finding several temperatures with maximum mass loss rate. There are 130 °C, 180 °C (the biggest), 240 °C, 320 °C, and 400 °C for powder mixture “Pyro” and 100 °C, 180 °C (the biggest), 230 °C, and 360 °C for powder mixture “Pyro_10Poly”. Mass loss due to CO_2_ (m/Z = 44) evolving took place in interval 110–500 °C with a maximum of 190 °C. Mass loss due to H_2_O (m/Z = 18) evolving took place in three intervals: 80–130 °C (with the maximum at 105 °C), 130–260 °C (with the maximum at 190 °C), 290–500 °C (with wide maximum 330–400 °C).

**Figure 4:**
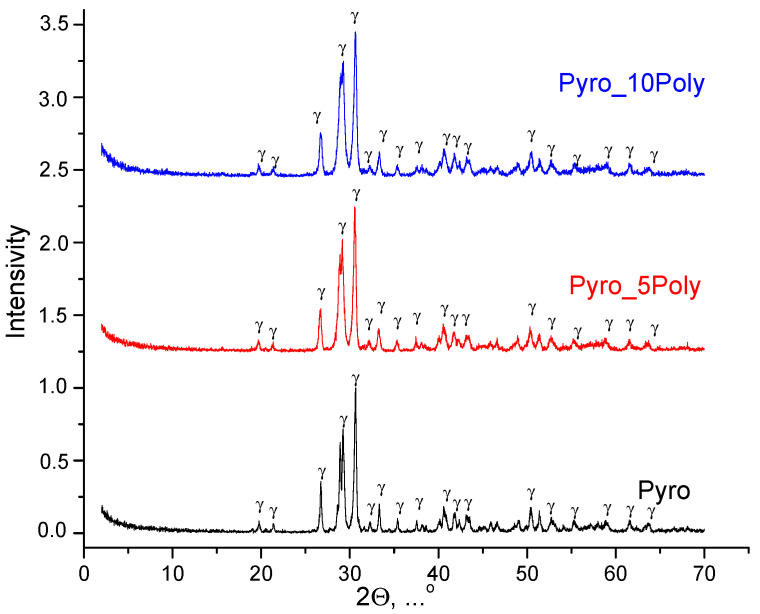
XRD data for powder mixtures prepared in mechanical activation conditions from calcium lactate pentahydrate Ca(C_3_H_5_O_3_)_2_·5H_2_O and monocalcium phosphate monohydrate Ca(H_2_PO_4_)_2_·H_2_O, disaggregated in acetone medium, after heat treatment at 600 °C: γ—γ-Ca_2_P_2_O_7_ (PDF card 17-499).

XRD data for powder mixtures prepared in mechanical activation conditions from calcium lactate pentahydrate Ca(C_3_H_5_O_3_)_2_·5H_2_O and monocalcium phosphate monohydrate Ca(H_2_PO_4_)_2_·H_2_O, disaggregated in acetone medium, after heat treatment at 600 °C is presented in Figure 4. The phase composition of prepared powders “Pyro”, “Pyro_05Poly”, “Pyro_10Poly” after heat treatment at 600 °C was presented by γ-calcium pyrophosphate γ-Ca_2_P_2_O_7_.

**Figure 5:**
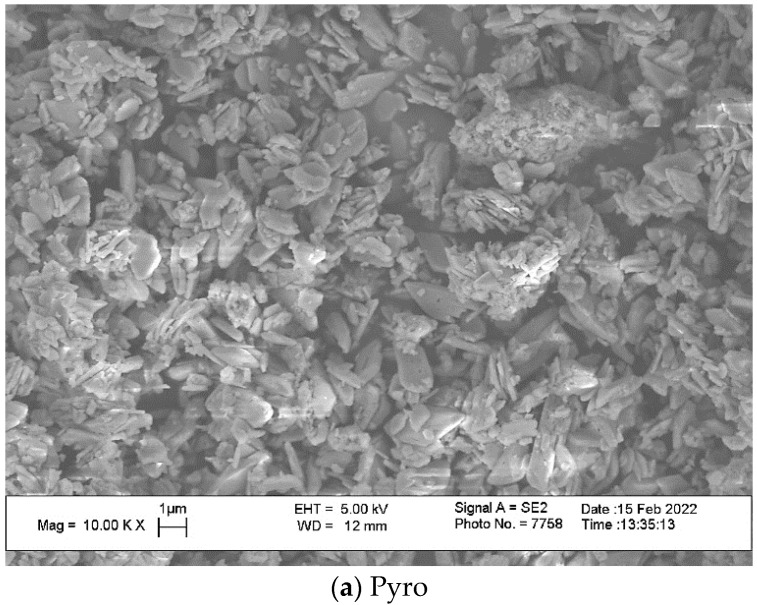

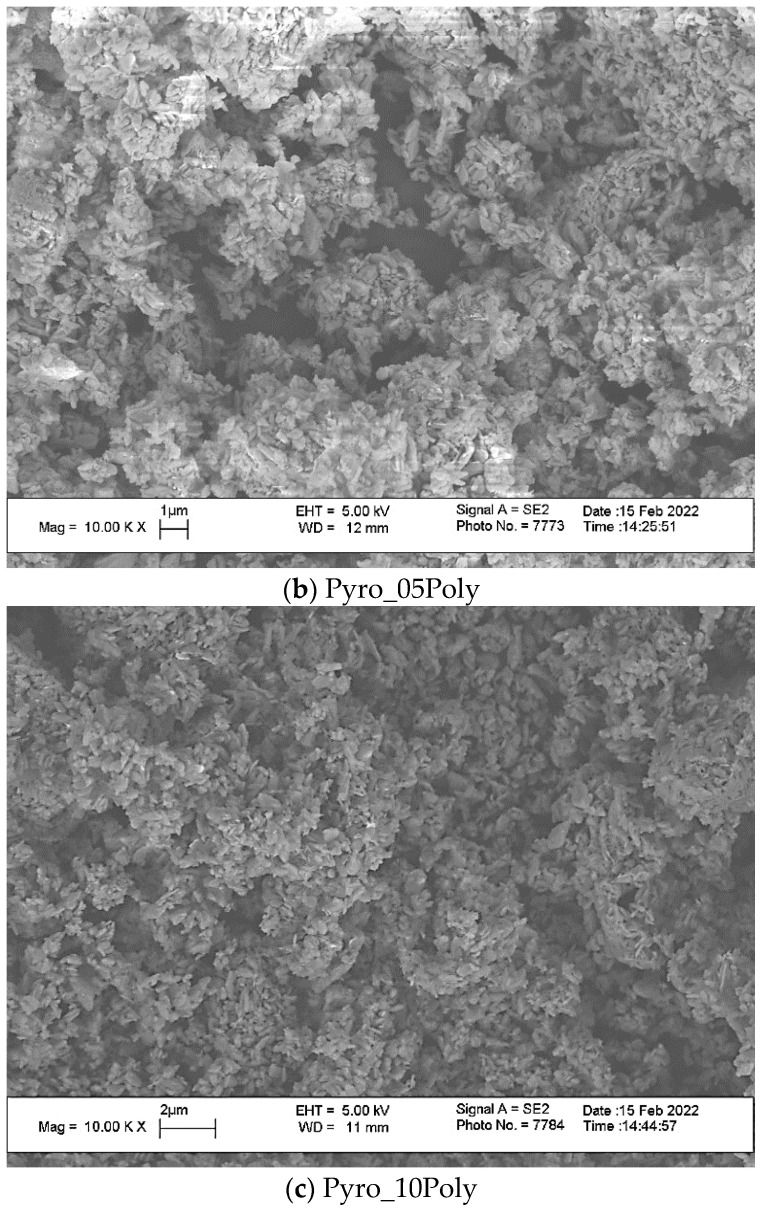
Micro photos of powders after heat treatment at 600 °C: “Pyro” (**a**); “Pyro_05Poly” (**b**); “Pyro_10Poly” (**c**).

SEM images of powders “Pyro”, “Pyro_05Poly”, “Pyro_10Poly” after heat treatment at 600 °C are presented in Figure 5. One can see that particles of powders are fine and the dimension of particles is dependent on the preset molar ratio of powders. Particles have a platelike morphology. Particles after heat treatment indeed inherit the shape of the particles after synthesis and drying. The lower the molar ratio Ca/P, the smaller the dimensions of particles are. The dimensions of particles of powder “Pyro” are in the interval 0.2–2 μm. The dimensions of particles of powder “Pyro_05Poly” are in interval 0.1–1 μm. The dimensions of particles of powder “Pyro_10Poly” are in the interval 0.1–0.5 μm. One of the reasons for fine particle formation consisted in using synthesis in the mechanical activation conditions. The advantages of using mechanical activation conditions were shown for monetite CaHPO_4_ synthesis before [10]. Presence of the lactic acid that acted as a surfactant [44] in suspensions during synthesis in mechanical activation conditions and during drying can be accepted as additional reason of fine particles formation. According to XRD analysis, brushite, the metastable dicalcium phosphate dihydrate CaHPO_4_·2H_2_O was detected only in the phase composition of the powder “Pyro”. Powders “Pyro_05Poly” and “Pyro_10Poly” contained monetite CaHPO_4_. All prepared powders after mechanical activation of starting components in water contained monocalcium phosphate monohydrate Ca(H_2_PO_4_)_2_·H_2_O. As all these calcium phosphates are slightly soluble in water to varying degrees, the following processes (reaction (10)–(13)) could take place during a week of drying of aqueous suspensions providing mass transfer between the particles of calcium phosphate phases.

CaHPO_4_⋅2H_2_O ⇄ Ca^2+^ + HPO_4_^2−^ + 2H_2_O (10)

CaHPO_4_ ⇄ Ca^2+^ + HPO_4_^2−^ (11)

Ca(H_2_PO_4_)_2_⋅H_2_O ⇄ Ca^2+^ + 2H_2_PO_4_^−^ + H_2_O (12)

2H_2_PO_4_^−^ ⇄ 2HPO_4_^2−^ + 2H^+^ (13)

Brushite CaHPO_4_⋅2H_2_O (*pK_sp_* = 6.59) is a more soluble salt than monetite CaHPO_4_ (*pK_sp_* = 6.90), and monocalcium phosphate monohydrate Ca(H_2_PO_4_)_2_⋅H_2_O (*pK_sp_* = 1.14) is significantly more soluble than brushite CaHPO_4_⋅2H_2_O and monetite CaHPO_4_ [2]. So, processes of dissolution/crystallization in suspension “Pyro” led to an increase in the particle size of brushite CaHPO_4_⋅2H_2_O during transformation of suspension to the powder. The lower the molar ratio of Ca/P in the powders from “Pyro” to “Pyro_10Poly”, the bigger the quantity of monocalcium phosphate monohydrate Ca(H_2_PO_4_)_2_⋅H_2_O in the water suspensions was. Therefore, the lower the preset molar ratio of Ca/P in samples under investigation, the greater the concentration of HPO_4_^2−^ and 2H^+^ ions in an aqueous solution surrounding calcium phosphate particles of suspensions were.

**Figure 6:**
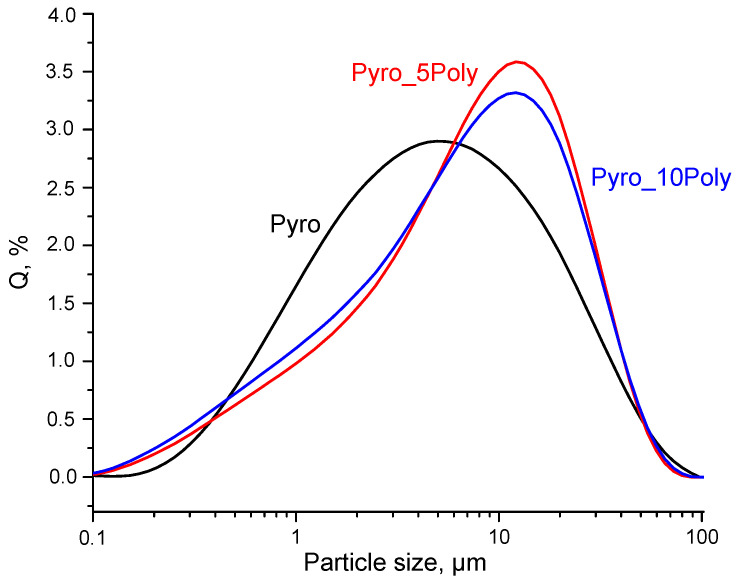
Particle size distribution of powders prepared in mechanical activation conditions from calcium lactate pentahydrate Ca(C_3_H_5_O_3_)_2_·5H_2_O and monocalcium phosphate monohydrate Ca(H_2_PO_4_)_2_·H_2_O, disaggregated in acetone medium, after heat treatment at 600 °C.

The bigger quantity of HPO_4_^2−^ and 2H^+^ ions in suspensions “Pyro_05Poly” and “Pyro_10Poly” could shift the equilibrium in reactions (10) and (11) to the left. This shift thereby reduced the recrystallization rate of solid phase particles of monetite CaHPO_4_. So, the possibility of dissolution/crystallization in suspension “Pyro” was more likely than in suspensions “Pyro_05Poly” and “Pyro_10Poly”. For this reason, the composition of prepared suspensions and the phenomenon of inheritance of synthesized particle morphology can explain the decrease in particle size in heat treated at 600 °C powders with a decrease in preset Ca/P molar ratio from 1 to 0.95. Reactions with fast, big volumes evolving of gaseous phase during heat treatment of powders could be the additional reason for fine powder formation. It should be noted that small particles of all powders are collected in aggregates. Particle size distribution of powder prepared in mechanical activation conditions from calcium lactate pentahydrate Ca(C_3_H_5_O_3_)_2_·5H_2_O and monocalcium phosphate monohydrate Ca(H_2_PO_4_)_2_·H_2_O, disaggregated in acetone medium, after heat treatment at 600 °C is presented in Figure 6. The size of most occurring aggregates of particles for powder “Pyro” estimated as 5.0 μm, for powder “Pyro_05Poly”-12.1 μm and for powder “Pyro_10Poly”-12.5 μm.

**Figure 7:**
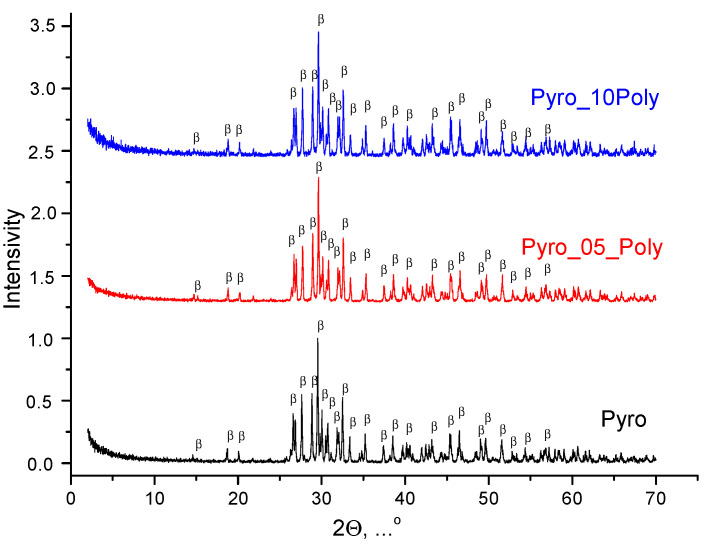
XRD data for ceramic samples “Pyro”, “Pyro_05Poly”, and “Pyro_10Poly” fired at 1100 °C: β—β-Ca_2_P_2_O_7_ (PDF card 9-346).

According to XRD data for ceramic samples based on powders “Pyro”, “Pyro_05Poly” and “Pyro_10Poly” fired at 900 °C, 1000 °C, and 1100 °C, the phase composition of all samples was presented by β-calcium pyrophosphate β-Ca_2_P_2_O_7_. XRD data for ceramic samples “Pyro”, “Pyro_05Poly”, “Pyro_10Poly” after firing at 900 °C, 1000 °C and 1100 °C can be found in Supplementary Materials at XRD data for ceramic samples after firing at 1100 °C are presented in Figure 7. So, we can conclude that presence of calcium polyphosphate Ca(PO_3_)_2_ up to 10 mol% introduced via excess of monocalcium phosphate monohydrate Ca(H_2_PO_4_)_2_·H_2_O cannot be detected using XRD analysis.

**Figure 8:**
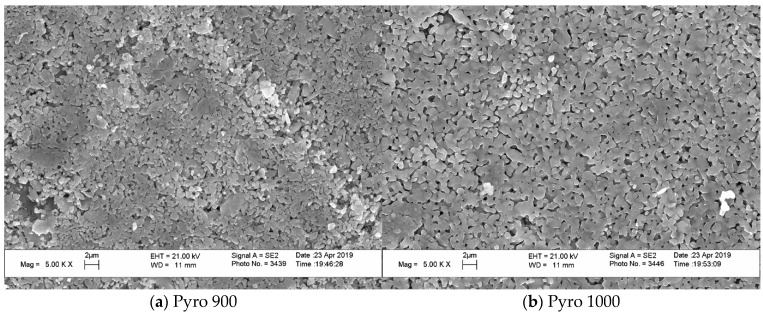

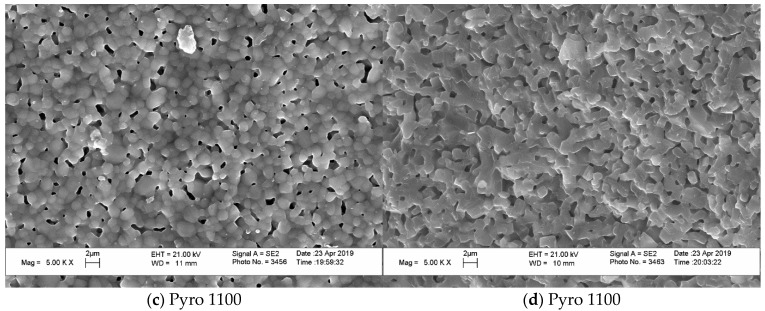
SEM micrographs of surface (**a**–**c**) and cross-section (**d**) of ceramic samples “Pyro” fired at 900 °C (**a**), 1000 °C (**b**), and 1100 °C (**c**,**d**).

**Figure 9:**
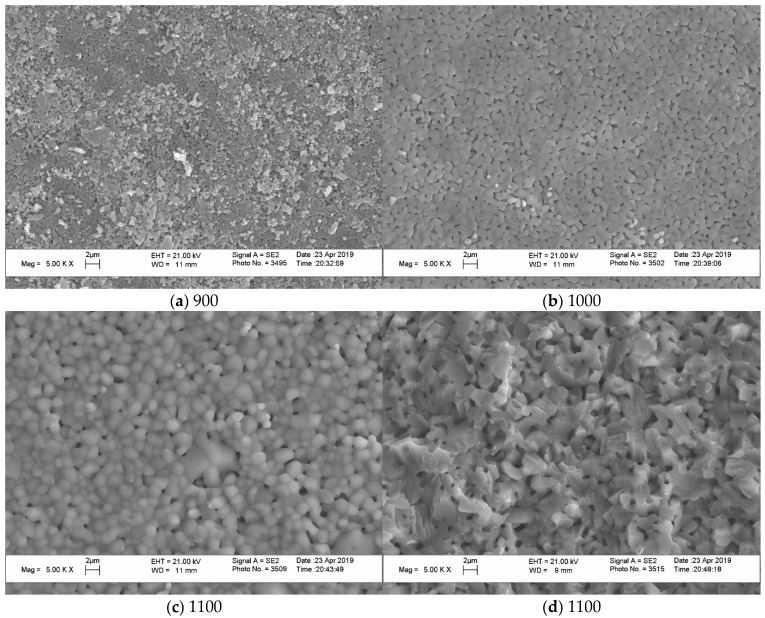
SEM micrographs of surface (**a**–**c**) and cross section (**d**) of ceramic samples “Pyro_05Poly” fired at 900 °C (**a**), 1000 °C (**b**) and 1100 °C (**c**,**d**).

**Figure 10:**
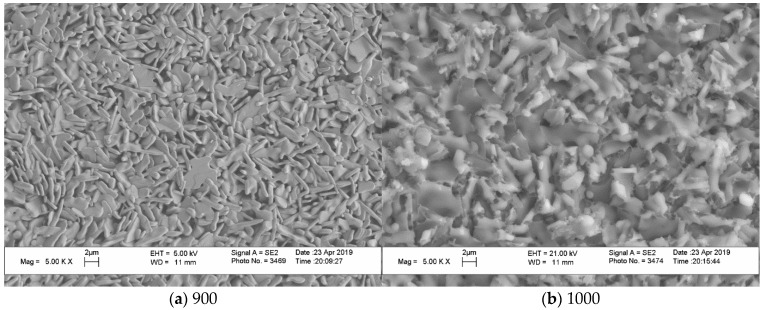

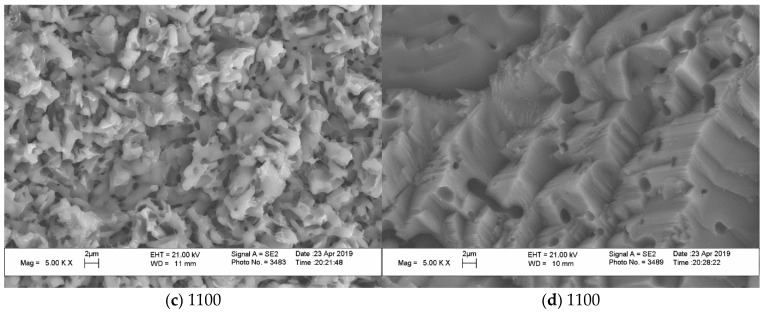
SEM micrographs of surface (**a**–**c**) and cross section (**d**) of ceramic samples “Pyro_10Poly” fired at 900 °C (**a**), 1000 °C (**b**), and 1100 °C (**c**,**d**).

At the same time, Figure 8 (“Pyro”), Figure 9 (“Pyro_05Poly”), and Figure 10 (“Pyro_10Poly”) present SEM micrographs of surface and cross-section of samples, allowing us to conclude the obvious influence of firing temperature and quantity of sintering additive on the microstructure of ceramics.

The grain size of ceramics increased both with the growth in firing temperature and with decreasing Ca/P molar ratio of powder mixtures. The grain size of ceramic samples based on powder “Pyro” (Figure 8) increased from 1 μm after firing at 900 °C (Figure 8a) to 2 μm after firing at 1100 °C (Figure 8c,d). It should be noted that impressions from the microstructure of surface and microstructure of cross-section after firing at 1100 °C are very much similar.

The grain size of ceramic samples based on powder “Pyro_05Poly” (Figure 9) increased from ~0.5–1 μm after firing at 900 °C (Figure 9a) to ~2 μm after firing at 1100 °C (Figure 9c,d). It should be noted that the sample despite the same development in grain size as it was for ceramics based on powder “Pyro” looks sintered to a greater extent. Microstructure of cross-section of the ceramic sample after firing at 1100 °C (Figure 9d) give us the opportunity to conclude that it formed at the presence of liquid phase. Temperatures of firing 1000 and 1100 °C are higher than eutectic temperature (970 °C) in the CaO-P_2_O_5_ system according to literature data [16]. So, the presence of calcium polyphosphate Ca(PO_3_)_2_ with the preset quantity of 5 mol.% creates the conditions for liquid phase sintering in ceramics based on powder “Pyro_05Poly”.

The microstructure of ceramics based on powder “Pyro_10Poly” (Figure 10) demonstrates the influence of additive provoking liquid phase sintering. One can see grains with dimensions 1–4 μm on the surface of the ceramic sample after firing at 900 °C (Figure 10a). Some grains have an elongated form. After firing at 1000 °C (Figure 10b), grains on the surface of the ceramic sample based on powder “Pyro_10Poly” have dimensions 2–4 μm; after firing at 1100 °C on the surface, one can see grains 2–4 μm (Figure 10c). Images of surfaces of ceramic samples bases on powder “Pyro_10Poly” after firing at 1000 °C and 1100 °C give us an opportunity to suppose that grains grew up in the direction above the surface. These phenomena were quite possible because the firing temperature 1000 °C and 1100 °C were higher than both the melting point (~984 °C) of calcium polyphosphate and eutectic point (~970 °C) in the quasi binary system Ca_2_P_2_O_7_-Ca(PO_3_)_2_ [16,54]. It is also well known that vapor pressure of polyphosphate melts is quite high [55]. Micrograph of cross-section (Figure 10d) of ceramics based on powder “Pyro_10Poly” after firing at 1100 °C does not show any grains and determination of their dimensions is not possible. One can see closed pores with dimensions 1–4 μm. The microstructure of cross-section gives us an opportunity to make a conclusion about presence of the melt in the ceramic sample based on powder “Pyro_10Poly” during firing at 1100 °C.

**Figure 11:**
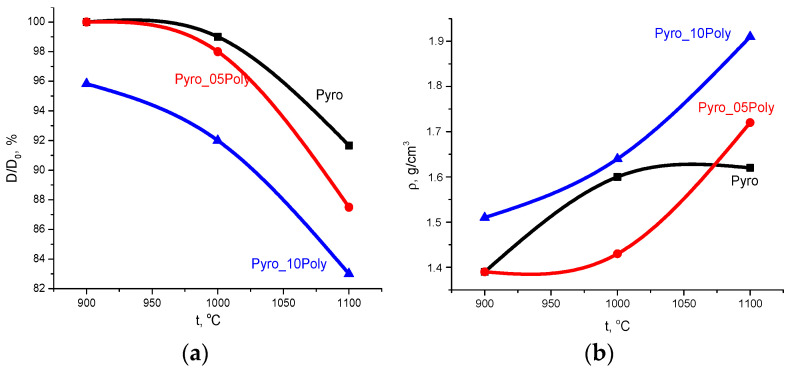
Dependences of relative diameter (**a**) and an apparent density (**b**) of ceramic samples from firing temperature.

Dependences of relative diameter (D/D_0_, %) and apparent density (g/cm^3^) of ceramic samples from firing temperature are presented in Figure 11.

Linear shrinkage (Figure 11a) increased for all samples with the growth in firing temperature. The maximum linear shrinkage of ceramics based on powder “Pyro” was ~10% after firing at 1100 °C. The maximum linear shrinkage of ceramics based on powder “Pyro_05Poly” was ~13% after firing at 1100 °C. The linear shrinkage of ceramics based on powder “Pyro_10Poly” increased from 4% at 900 °C to 17% at 1100 °C.

Density of ceramic samples (Figure 11b) prepared from the powders “Pyro_05Poly” and “Pyro_10Poly” increased with growth in firing temperature from 1.4 g/cm^3^ and 1.51 g/cm^3^ at 900 °C to 1.7 g/cm^3^ (55%) and 1.9 g/cm^3^ (60%) at 1100 °C, respectively. The density of ceramic samples based on powders “Pyro” achieved 1.6 g/cm^3^ (50%) after firing at 1000 °C; after firing at 1100 °C, this value became the same. In comparison with theoretical density of β-calcium pyrophosphate (3.12 g/cm^3^), we have to admit that as a result, we prepared quite porous ceramic samples from fine powders of γ-calcium pyrophosphate.

**Figure 12:**
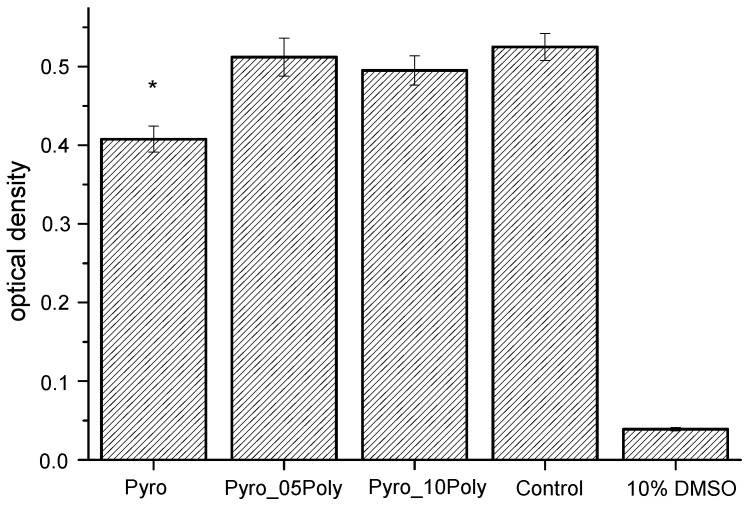
The MTT viability assay of NCTC L929 cells in the presence of liquid extracts from ceramic samples (firing temperature 1100 °C) under investigation, i.e., “Pyro”, “Pyro_05Poly”, “Pyro_10Poly”, control, and 10% DMSO after 48 h cultivation (mean ± SD, n = 10).

The results of the MTT-test are presented in Figure 12. The MTT assay showed the viability assay of NCTC L929 cells in the presence of liquid extracts from ceramic samples under investigation, i.e., “Pyro”, “Pyro_05Poly”, and “Pyro_10Poly” after 48 h cultivation sample and control. The Mann–Whitney U test was performed to assess the significance of the effect of liquid extracts from ceramic samples under investigation i.e., “Pyro”, “Pyro_05Poly”, and “Pyro_10Poly”, on the cell viability assay. There was no significant difference between the groups “Pyro_05 Poly” and “Pyro_10 Poly” when comparing control. The effect of liquid extracts from ceramic samples “Pyro” (*) obtained results is significantly different from the control sample.

**Figure 13:**
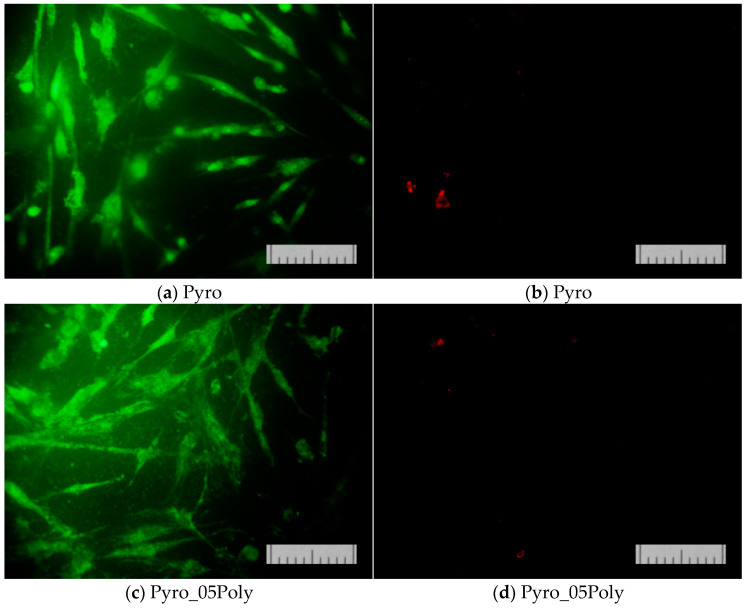

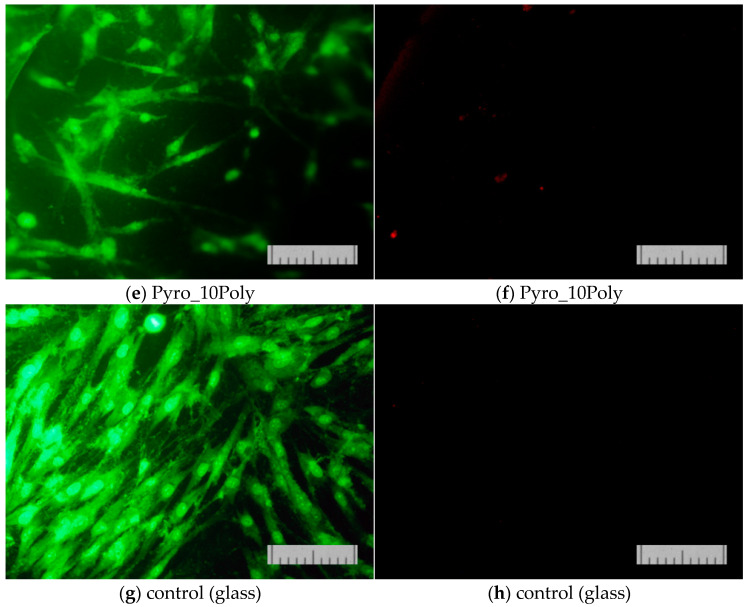
The appearance of the dental pulp stem cells on the surface of ceramic samples (firing temperature 1100 °C) under investigation, i.e., ceramic samples prepared based on powders “Pyro” (**a**,**b**), “Pyro_05Poly” (**c**,**d**), “Pyro_10Poly” (**e**,**f**), and control (**g**,**h**) after direct contact procedure for 2 days. Fluorescent staining was made with SYTO 9 (**a**,**c**,**e**,**g**) and propidium iodide (**b**,**d**,**f**,**h**). Bar 100 μm.

**Figure 14:**
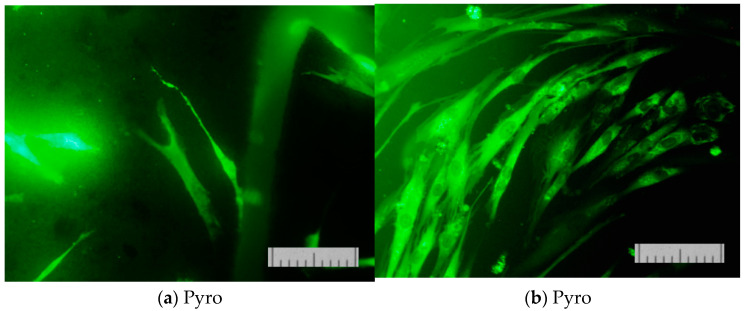

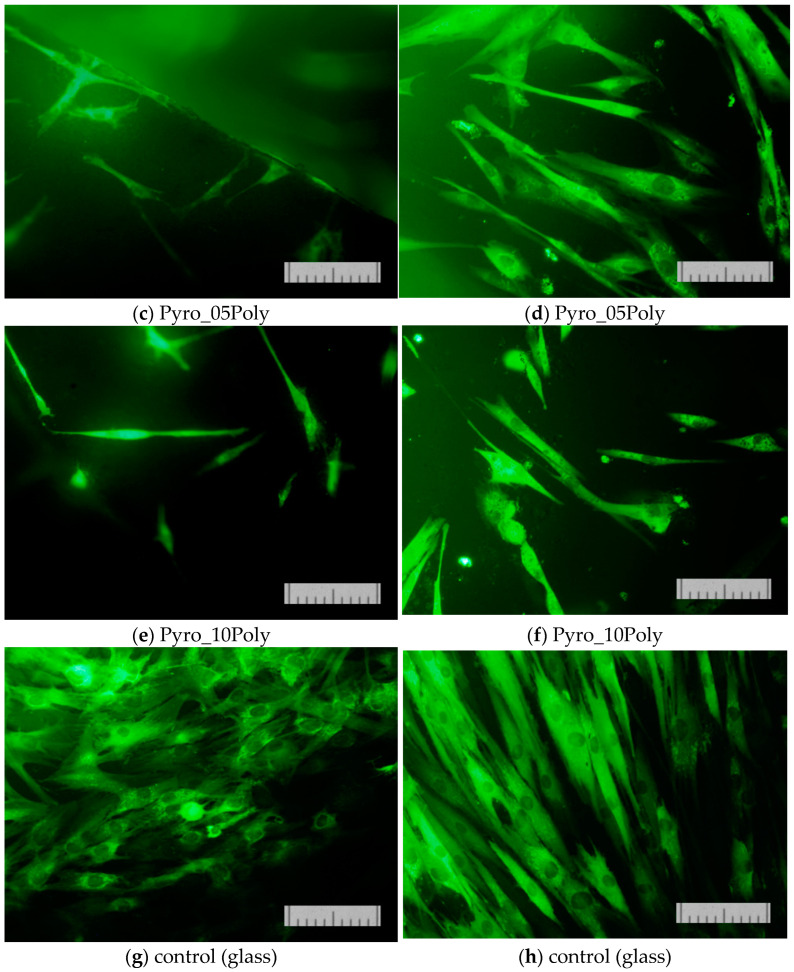
The appearance of the dental pulp stem cells of the surface of ceramic samples (firing temperature 1100 °C) under investigation, i.e., “Pyro” (**a**,**b**), “Pyro_05Poly” (**c**,**d**), “Pyro_10Poly” (**e**,**f**), and control (**g**,**h**) after direct contact procedure for 2 (**a**,**c**,**e**,**g**) and 7 (**b**,**d**,**f**,**h**) days. Fluorescent staining was made with SYTO 9. Bar—100 μm.

The results of determining the viability of cells cultured on the surface of the studied materials on the second (Figure 13) and on the second and seventh day (Figure 14) confirmed that the proliferative activity of cells was observed on the surface of all the samples studied.

Normal morphology of DPSC 32 cells is observed on all the studied samples. However, the density of the cell layer on the surface of the studied samples after cultivation for two or seven days was slightly lower than in the control (on the cover glass). This phenomenon was apparently due to the conditions of initial cell adhesion. Nevertheless, the absence of dead cells whose nuclei are stained with propidium iodide indicates the absence of cytotoxic effects of the ceramic materials prepared based on powders “Pyro”, “Pyro_05Poly”, and “Pyro_10Poly”. The density of the cell layer on the surface of the studied samples after cultivation for seven days (Figure 14b,d,f) demonstrate the slight dependence from preset Ca/P molar ratios in starting powders and ceramic samples. The lower the Ca/P molar ratio, the lower the density of cell layer. This phenomenon can be explained with the more acidic nature of ceramic samples (“Pyro_05Poly”, “Pyro_10Poly”) containing the calcium polyphosphate phase in a very slight quantity not detected by XRD analysis.

**Figure 15:**
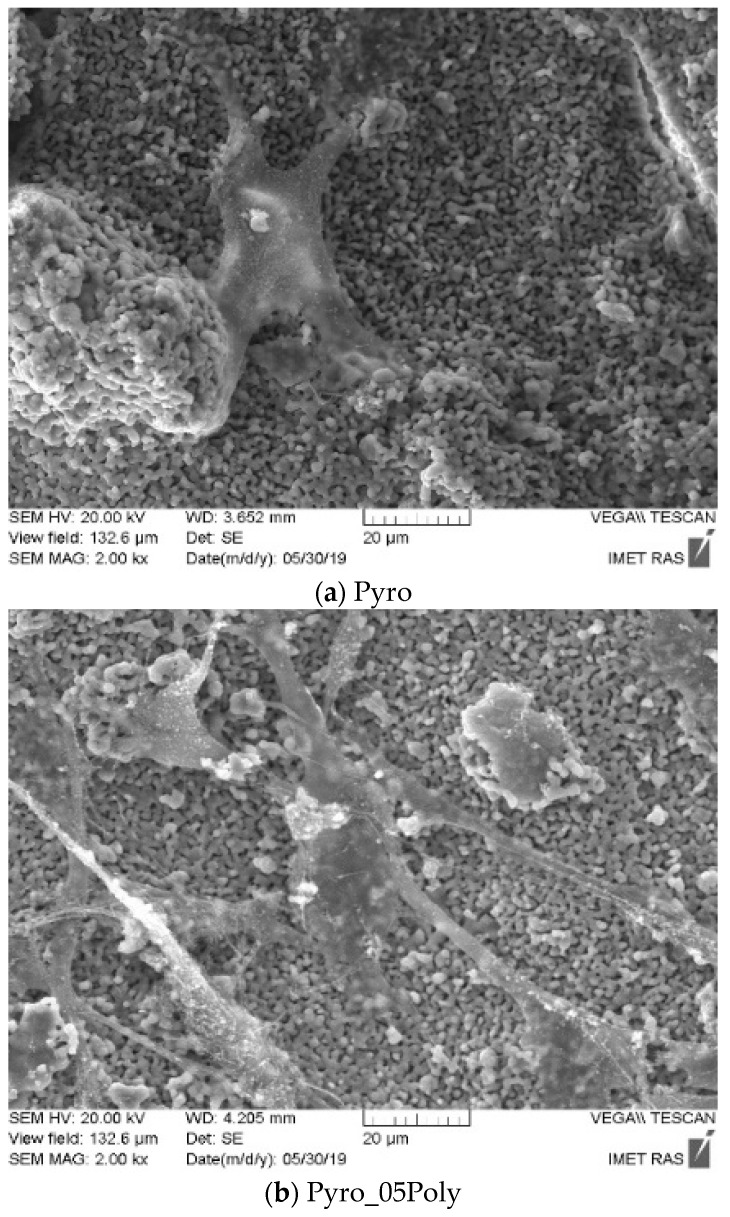

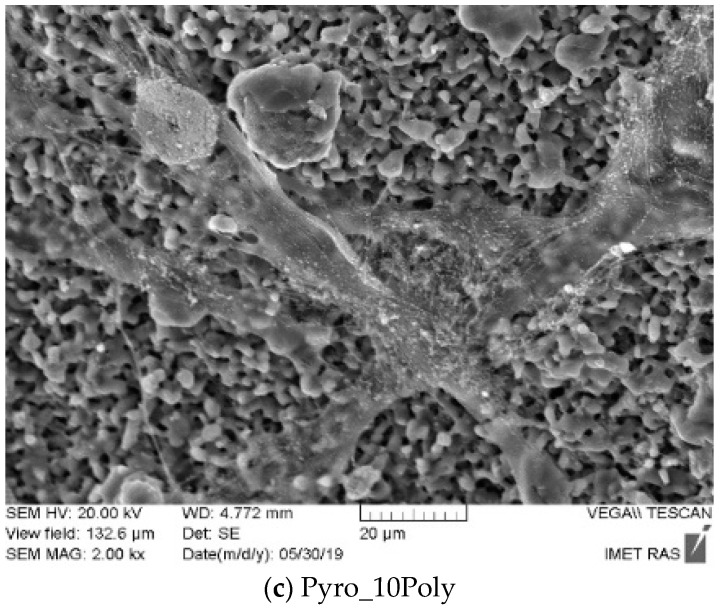
SEM images of cells on the surface of ceramic samples (firing temperature 1100 °C) under investigation, i.e., “Pyro” (**a**), “Pyro_05Poly” (**b**), “Pyro_10Poly” (**c**) after 2 days of cultivation.

Figure 15 presents micrographs of cells fixed to the ceramic surface after cultivation for two days.

These images (Figure 15) demonstrate the good adhesion of cells on the surface of β-calcium pyrophosphate β-Ca_2_P_2_O_7_ ceramic samples prepared based on powders “Pyro” (a), “Pyro_05Poly” (b), and “Pyro_10Poly” (c).

Data from in vitro biological experiments confirmed the biocompatibility of the obtained β-calcium pyrophosphate β-Ca_2_P_2_O_7_ ceramic materials and their ability to support cells proliferation.

## 4. Conclusions

The original method of γ-calcium pyrophosphate γ-Ca_2_P_2_O_7_ powder preparation was used. To prepare powders of γ-calcium pyrophosphate γ-Ca_2_P_2_O_7_ with preset molar ratios Ca/P = 1, 0.975, and 0.95 powder mixtures based on calcium lactate pentahydrate Ca(C_3_H_5_O_3_)_2_·5H_2_O and monocalcium phosphate monohydrate Ca(H_2_PO_4_)_2_·H_2_O were treated in an aqua medium in mechanical activation conditions, dried, disaggregated in acetone, and heat-treated at 600 °C. The addition of more excess of monocalcium phosphate monohydrate Ca(H_2_PO_4_)_2_·H_2_O (with appropriate molar ratio of Ca/P = 1) to the mixture of starting components resulted in lower dimensions of γ-calcium pyrophosphate γ-Ca_2_P_2_O_7_ individual particles. Porous ceramic samples with the relative density of 50% (“Pyro”), 55% (“Pyro_05Poly”), and 60% (“Pyro_10Poly”) in the CaO-P_2_O_5_ system were created from prepared powders after firing at 1100 °C. The grain size of ceramic samples increased both with the growth in firing temperature and with decreasing molar ratio Ca/P of powder mixtures. Calcium polyphosphate (t _melt_ =984 °C), which formed from monocalcium phosphate monohydrate Ca(H_2_PO_4_)_2_·H_2_O, acted similar to a liquid phase sintering additive. It was confirmed by tests in vitro, that prepared ceramic materials with preset molar ratios Ca/P = 1, 0.975, and 0.95 and phase composition presented by β-calcium pyrophosphate β-Ca_2_P_2_O_7_ were biocompatible and could maintain bone cells proliferation.

## Figures and Tables

**Table 1:** Description of target phase composition of ceramics.

Sample	Ca/P Molar Ratio	The Phase Composition, mol.%		The Phase Composition, mas.%	
		β-Ca_2_P_2_O_7_	β-Ca(PO_3_)_2_	β-Ca_2_P_2_O_7_	β-Ca(PO_3_)_2_
Pyro	1	100	0	100	0
Pyro_05Poly	0.975	95	5	96	4
Pyro_10Poly	0.95	90	10	92	8

**Table 2:** Composition of powder mixtures before treatment in mechanical activation conditions.

Sample	Ca/P Molar Ratio	Starting Components, mol.%		Starting Components, mas.%	
		Ca(C_3_H_5_O_3_)_2_⋅5H_2_O	Ca(H_2_PO_4_)_2_⋅H_2_O	Ca(C_3_H_5_O_3_)_2_⋅5H_2_O	Ca(H_2_PO_4_)_2_⋅H_2_O
Pyro	1	50.0	50.0	55.0	45.0
Pyro_05Poly	0.975	48.7	51.3	53.7	46.3
Pyro_10Poly	0.95	47.4	52.6	52.4	47.6

## Data Availability

Not applicable.

## Supplementary Materials

The following supporting information can be downloaded at: https://www.mdpi.com/article/10.3390/ma15093105/s1. Figure S1: (XRD data for ceramic samples “Pyro” fired at 900 °C, 1000 °C and 1100 °C: β—β-Ca_2_P_2_O_7_ (PDF card 9-346)); Figure S2: (XRD data for ceramic samples “Pyro_05Poly” fired at 900 °C, 1000 °C and 1100 °C: β—β-Ca_2_P_2_O_7_ (PDF card 9-346)); Figure S3: (XRD data for ceramic samples “Pyro_10Poly” fired at 900 °C, 1000 °C, and 1100 °C: β—β-Ca_2_P_2_O_7_ (PDF card 9-346)).
