# Supplementary material for: Bioceramics Based on β-Calcium Pyrophosphate
Source: Materials (Basel). 2022 Apr 25;15(9):3105. doi: 10.3390/ma15093105 (PMC9101783; doi:10.3390/ma15093105)
Supplement: Supplementary file 1 [file materials-15-03105-s001.zip › materials-1626578-supplementary.pdf]

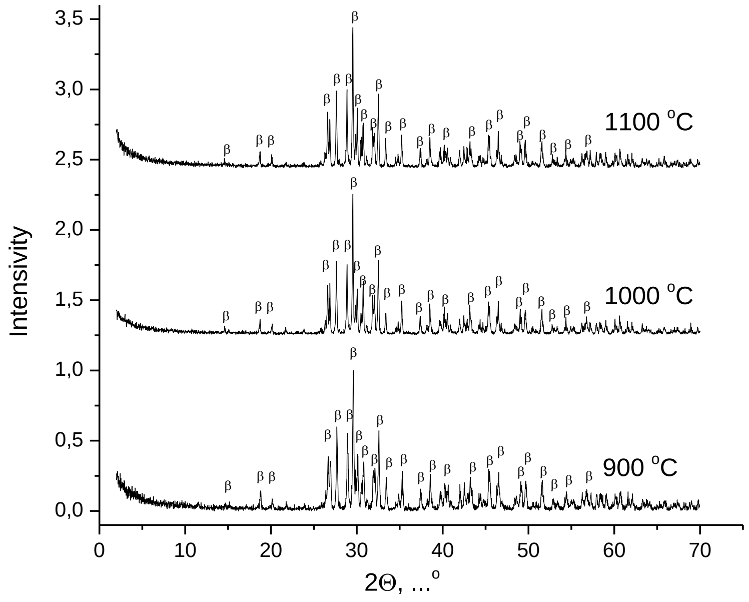

Figure S1. XRD data for ceramic samples "Pyro" fired at 900 °C, 1000 °C and 1100 °C: β – β-$Ca_2P_2O_7$ (PDF card 9-346).

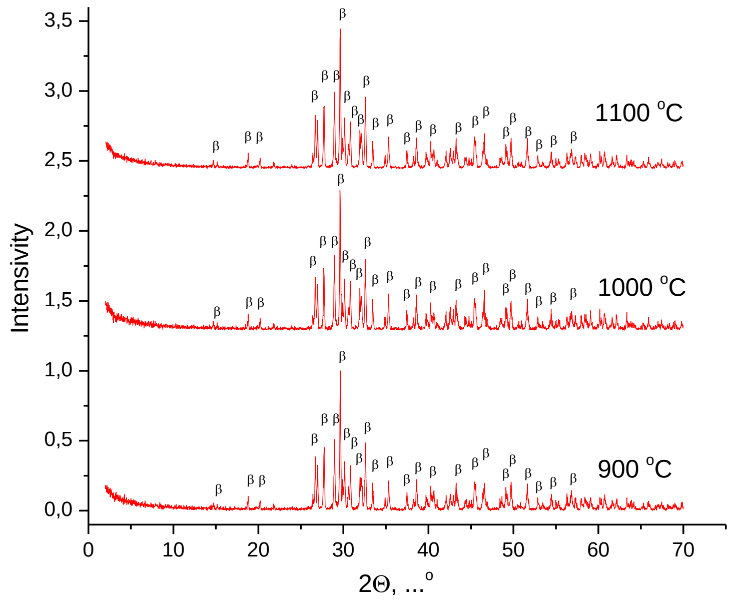

Figure S2. XRD data for ceramic samples "Pyro_05Poly" fired at 900 °C, 1000 °C and 1100 °C: β – β-$Ca_2P_2O_7$ (PDF card 9-346).

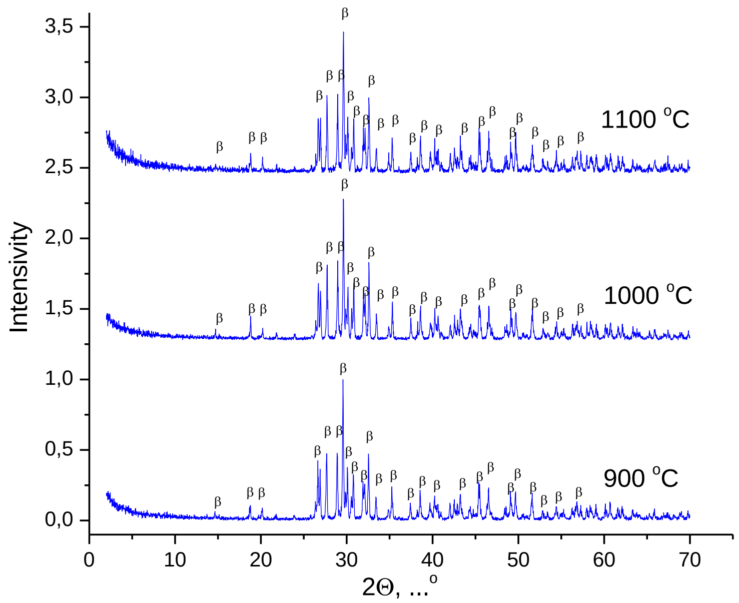

Figure S3. XRD data for ceramic samples "Pyro_10Poly" fired at 900 °C, 1000 °C, and 1100 °C: β – β-$Ca_2P_2O_7$ (PDF card 9-346).
